# Positron Emission Tomography in Heart Failure: From Pathophysiology to Clinical Application

**DOI:** 10.3390/jcdd10050220

**Published:** 2023-05-17

**Authors:** Gregorio Tersalvi, Vittorio Beltrani, Martin R. Grübler, Alessandra Molteni, Yvonne Cristoforetti, Giovanni Pedrazzini, Giorgio Treglia, Luigi Biasco

**Affiliations:** 1 Department of Cardiology, Cardiocentro Ticino Institute, Ente Ospedaliero Cantonale, 6900 Lugano, Switzerland; 2 Department of Internal Medicine, Ente Ospedaliero Cantonale, 6850 Mendrisio, Switzerland; 3 Department of Cardiology, Regional Hospital Neustadt, 2700 Wiener Neustadt, Austria; 4 Department of Internal Medicine, Medical University of Graz, 8036 Graz, Austria; 5 Faculty of Biomedical Sciences, Università della Svizzera Italiana (USI), 6900 Lugano, Switzerland; 6 Clinic of Nuclear Medicine, Imaging Institute of Southern Switzerland, Ente Ospedaliero Cantonale, 6500 Bellinzona, Switzerland; 7 Faculty of Biology and Medicine, University of Lausanne (UNIL), 1015 Lausanne, Switzerland; 8 Division of Cardiology, Azienda Sanitaria Locale Torino 4, 10073 Ospedale di Ciriè, Italy

**Keywords:** PET, radionuclide, microvascular dysfunction, metabolic imaging, heart failure

## Abstract

Imaging modalities are increasingly being used to evaluate the underlying pathophysiology of heart failure. Positron emission tomography (PET) is a non-invasive imaging technique that uses radioactive tracers to visualize and measure biological processes in vivo. PET imaging of the heart uses different radiopharmaceuticals to provide information on myocardial metabolism, perfusion, inflammation, fibrosis, and sympathetic nervous system activity, which are all important contributors to the development and progression of heart failure. This narrative review provides an overview of the use of PET imaging in heart failure, highlighting the different PET tracers and modalities, and discussing fields of present and future clinical application.

## 1. Introduction

Heart failure (HF) affects millions of people worldwide, causing significant morbidity and mortality [1]. The diagnosis of HF is primarily based on clinical symptoms and physical examination, but imaging modalities are increasingly being used to evaluate the underlying pathophysiology of the disease.

**Figure 1:**
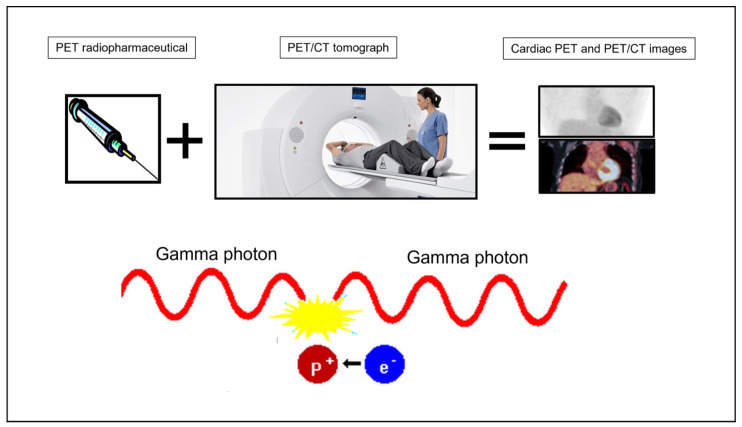
Operating principle of cardiac PET.

Positron emission tomography (PET) is a non-invasive imaging technique that uses radioactive tracers in which a compound or pharmaceutical is labeled with a positron-emitting radionuclide. A positron is a positively charged nuclear particle that has the same mass as an electron. When an emitted positron collides with an electron, an annihilation reaction occurs, producing two gamma photons in opposite directions (Figure 1) [2]. PET detectors only register photon pairs that hit opposing detectors simultaneously. PET radionuclides generally have much shorter half-lives than those used in single photon emission computed tomography (SPECT), thus having less radiation exposure [2,3]. Furthermore, there is an advantage in terms of the spatial resolution of PET compared to SPECT [3]. PET imaging can be used to quantify the absolute number of radiopharmaceuticals within the myocardium. Beyond static PET images, the development of dynamic PET images can be used to assess tracer kinetics within the myocardium [4].

PET imaging of the heart using different radiotracers can provide information on myocardial metabolism, perfusion, fibrosis, and sympathetic nervous system activity, which are all important contributors to the development and progression of HF. Different pathophysiological mechanisms of HF are assessed with different radiopharmaceuticals (Table 1). The most commonly utilized cardiac PET tracers include molecules to assess myocardial blood flow (MBF), such as ^13^N–Ammonia ([^13^N]H_3_), Rubidium-82 (^82^Rb), oxygen-15 labeled water (H_2_[^15^O]), and [^18^F]Flurpiridaz, or myocardial metabolism, such as fluorine–18 fluorodeoxyglucose ([^18^F]FDG) and [^11^C]acetate [2].

Originally, PET imaging has been established in the evaluation of coronary disease since it offers myocardial viability assessment and quantification of regional MBF [25,26]. Beyond this role, PET imaging has been shown to also provide valuable information in patients with valvular heart disease, sarcoidosis, amyloidosis, and other forms of cardiomyopathy [27]. In the experimental field of HF, PET has been used to highlight different pathways of myocardial energetics, inflammation, and structural remodeling, both in human and animal models [16,28,29].

This review provides an overview of the use of PET imaging in HF, highlighting the different PET tracers and modalities and discussing fields of present and future clinical application.

## 2. Pathophysiology

**Figure 2:**
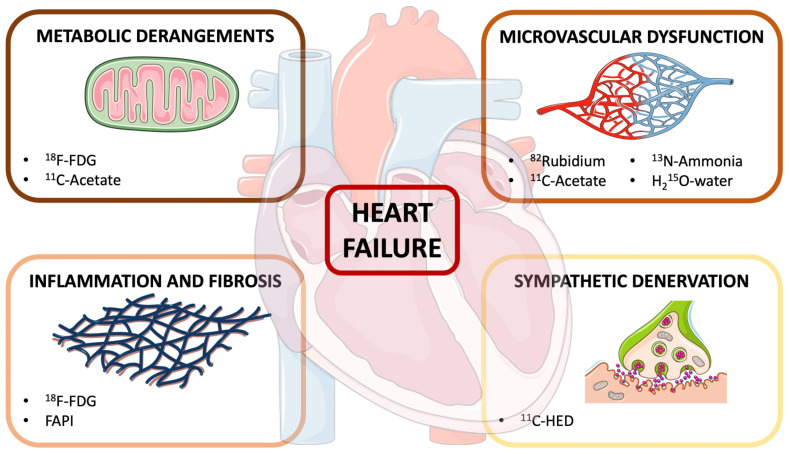
Pathophysiological mechanisms of heart failure and corresponding PET tracers. Modified from Server Medical Art (licensed under a Creative Common Attribution 3.0 Generic License. http://smart.servier.com/ (accessed on 23 March 2023)).

Each HF phenotype accounts for a peculiar underlying pathophysiological mechanism. As an example, ischemic HF with reduced ejection fraction (HFrEF) is prevalently associated with scarring, eccentric remodeling, and left ventricular (LV) dilatation, whilst HF with preserved ejection fraction (HFpEF) mainly relies on metabolic comorbidities, inflammation, and diastolic dysfunction [30,31,32,33]. However, shared pathways are involved in the development of HF irrespective of LV ejection fraction (LVEF). These include metabolic derangements, microvascular dysfunction, inflammation, fibrosis, and sympathetic dysfunction. PET imaging might reveal the activation of these peculiar biological processes in HF using different tracers (Figure 2).

### 2.1. Metabolic Derangements

The myocardium has a massive energy requirement, consuming more energy and oxygen than any other organ [34]. It continuously produces substantial amounts of adenosine triphosphate (ATP), which is essential for maintaining active myocardial contraction and diastolic relaxation [35]. To achieve this, the myocardium must have metabolic fuel flexibility, allowing it to use a variety of substrates, such as fatty acids, carbohydrates, ketones, and amino acids, to generate the donors for mitochondrial electron transport and ATP production [36]. This metabolic adaptability enables the heart to adjust to acute stressors. In the failing heart, myocardial fatty acid uptake rates are higher than expected for the normal heart, whereas myocardial glucose uptake rates are lower. This shift in myocardial substrate use may be an indication of impaired energy efficiency in HF [37]. In general, prolonged abnormalities in multiple metabolic pathways prevent the heart from sustaining the necessary levels of ATP for cardiac function, ultimately contributing to HF [38].

The traditional way to assess cardiac substrate utilization is by measuring the arteriovenous differences in oxygen, glucose, and fatty acids, together with coronary blood flow by means of invasive simultaneous arterial and coronary sinus blood sampling. PET imaging has allowed non-invasive measurement of fatty acids and glucose utilization by following the uptake of tracers, e.g., [^11^C]acetate and fluorine-18-fluoro-6-thia-heptadecanoic acid ([18F]FTHA) for the measurement of fatty acids metabolism and [^18^F]FDG for the measurement of glucose metabolism [4]. As acetate is readily taken up and oxidized via the tricarboxylic acid cycle, [^11^C]acetate serves as a measure of oxidative metabolism and, indirectly, of myocardial oxygen consumption (MVO_2_) [28]. In contrast, [^18^F]FDG measures the uptake and initial conversion step, rather than the oxidation of glucose and fatty acids [39]. FDG works as a glucose analog that competes with glucose for trans-membranous transport sites. Thus, it traces the initial phosphorylation of glucose to glucose-6-phosphate and is a quantitative marker for the rate of exogenous glucose utilization in the myocardium. However, since FDG is a poor substrate for glycolysis, glycogen synthesis, and pentose phosphate shunt pathways, and combined with the fact that dephosphorylation of FDG-6-phosphate is slow, FDG-6-phosphate accumulates in the cardiomyocyte. In activated inflammatory cells, high levels of glucose transporters are expressed and thus increased 18F-FDG uptake is present if the remaining, supposedly healthy, myocardium is adequately suppressed. Therefore, FDG kinetics allow conclusions in regard to perfusion, hibernation, and inflammation [40].

A study estimating regional myocardial fatty acid myocardial utilization with [^11^C]palmitate showed significant visual differentiation between ischemic and non-ischemic causes of HF, reporting 80% sensitivity and 100% specificity [41]. Another study using [^13^N]H_3_ PET for perfusion and [^18^F]FDG PET for glucose metabolism reported 100% sensitivity and 80% specificity for HF differentiation (ischemic vs. non-ischemic) [42].

The use of PET imaging to evaluate cardiomyocyte metabolism can be extended to the early detection of chemotherapy-induced HF [43], which is discussed in the paragraph “Clinical Applications”.

### 2.2. Microvascular Dysfunction

The endothelium is characterized by endocrine and paracrine activities, which regulate vascular function and structure [44]. In the healthy endothelium, different stimuli mediated by receptors and blood flow can activate the production and release of nitric oxide. The latter induces relaxation of the vascular smooth muscle cells, vasodilation, platelet aggregation inhibition, and anti-inflammatory effects [45,46].

Endothelium-dependent vasodilation is dampened both in hFrEF and in hFpEF [47]. The endothelial dysregulation in HF patients is likely due to increased formation of superoxide radicals and other oxidant species. In general, conditions producing oxidative stress alter the balance between oxygen free radicals formation and their inactivation through endogenous antioxidant systems, causing direct inactivation of nitric oxide, with subsequent deterioration of endothelial and microvascular function [45,46,48]. The importance of coronary vasodilation in meeting the increased demand for energy substrate and oxygen by the myocardium should be underlined. Unlike most other tissues, where increased demand can be met by increasing extraction of oxygen and nutrients from the circulation, in the myocardium, extraction at rest is maximum and any increase in demand can only be satisfied with vasodilatation to increase myocardial blood flow.

PET is regarded as the gold standard for the quantification of perfusion and offers insights into the phenotypes of microvascular dysfunction (MVD) through the assessment of MBF or myocardial perfusion reserve (MPR) [49]. The latter, defined as the ratio of global MBF at stress versus at rest [50], is a surrogate measure of the vasodilatory capacity of small vessels and an accepted proxy for MVD after the exclusion of epicardial coronary artery disease (CAD) [51].

Abnormal MPR, assessed with Rubidium-82 (^82^Rb) PET, is associated with cardiovascular outcomes such as cardiac death, nonfatal myocardial infarction (MI), revascularization, and HF hospitalizations [52]. In a large cohort of subjects with preserved LVEF, in which global and regional MPR were assessed using ^82^Rb PET [49], mean MPR was significantly lower in HFpEF patients compared to both hypertensive and normotensive subjects without HF. MVD, defined as MPR < 2, was present in 40% of hFpEF patients. hFpEF was associated with 2.62-fold greater unadjusted odds of having global impairment in MPR and remained a significant predictor of reduced global MPR after adjusting for major comorbidities [49].

In another study using ^82^Rb PET, decreased MPR was associated with diastolic dysfunction, increased filling pressures, and abnormal LA strain in patients with preserved ejection fraction, supporting the hypothesis that MVD contributes to cardiac functional alterations observed in hFpEF [53]. For the diagnosis of microvascular angina, PET is currently the most validated non-invasive imaging modality [54]. Fewer data are available for quantitative perfusion in cardiac magnetic resonance (CMR), but clinical guidelines do not currently recommend one above the other imaging modality [55].

Although ^82^Rb is the preferred tracer at most PET sites, the gold standard for quantitative measurement of MBF and MPR is H_2_[^15^O]. However, the ^15^O-isotope has a short half-life of about two minutes, requiring an onsite cyclotron for its production, thus limiting its use [56].

A recent study with [^11^C]acetate PET imaging characterized LV external work, MVO_2_, and MBF in patients with HFpEF compared to age/sex-matched healthy controls [28]. During dobutamine stress, external work, MVO_2_, and MBF increased in both HFpEF and controls. However, the magnitude of increases was significantly smaller in HFpEF. In both groups, MBF increased in relation to external work, but in HFpEF, the slope of the relationship was significantly smaller than in controls [28].

In summary, PET imaging with different tracers such as ^82^Rb and [^11^C]acetate can help characterize patients with MVD and abnormal MPR, especially in the spectrum of HFpEF. Further studies are needed to assess whether measures of MVD can be used to assess disease progression in HF or, indeed, whether MVD is a potential treatment target [57].

### 2.3. Inflammation and Fibrosis

Systemic inflammation has been recognized as a common feature of all HF subtypes [58,59]. Inflammatory cytokines play a major role in myocyte stress or stretch, myocyte injury and apoptosis, fibroblast activation, and extracellular matrix remodeling, and have thus been extensively studied in patients with HF [60]. Inflammation is associated with disease development, progression, and major complications, and is predictive of poor outcomes independent of traditional metrics such as LVEF or New York Heart Association (NYHA) functional class [61].

**Figure 3:**
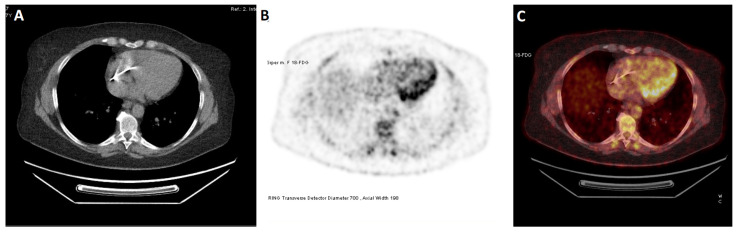
Axial [^18^F]FDG PET/CT showing increased uptake of [^18^F]FDG in the lateral wall in a female patient with cardiac sarcoidosis. Notably, myocardial inflammation at [^18^F]FDG PET may be difficult to distinguish from incomplete suppression of myocardial glucose utilization. Correlation with myocardial perfusion imaging, as well as clinical features and other imaging modalities, may be useful in this regard. In this case, the treatment decision was based on multiple factors, including scarring on cardiac MRI, positive lymph node biopsy, and increasing burden of ventricular arrhythmias. (**A**) CT; (**B**) PET; (**C**) PET/CT.

The paradigm of myocardial inflammatory disease for which PET imaging has gained a major role is cardiac sarcoidosis (Figure 3). The great spatial resolution of traditional myocardial perfusion imaging with PET coupled with its ability to identify abnormal [^18^F]FDG uptake has made it feasible to identify a pattern of mismatch between perfusion and metabolism suggestive of active cardiac sarcoidosis [22,62]. Other imaging modalities, especially echocardiography, lack the ability to reliably detect myocardial inflammation. Even CMR with T2 mapping shows only a moderate correlation with PET images [63,64]. Further data regarding PET imaging in cardiac sarcoidosis are discussed in the paragraph “Clinical Applications”.

In myocarditis, where CMR is considered the non-invasive gold standard, recent evidence suggests that [^18^F]FDG PET may improve sensitivity [65]. Whether this observation relates to different types of myocarditis (chronic vs. acute) remains to be elucidated.

Recently, [^18^F]FDG PET imaging assessing myocardial glucose metabolism has been used to identify inflammation in ischemic heart disease [66]. Overload pressure rapidly increases acute inflammatory cell infiltration with rising [^18^F]FDG uptake in the myocardium [67]. Nevertheless, [^18^F]FDG imaging could not effectively distinguish inflammatory cells in the presence of extensive cardiomyocyte metabolic remodeling due to pressure overload [67]. Furthermore, [^18^F]FDG PET/computed tomography (CT) visualizes inflammatory reactions, but not cardiac fibrosis activation. Thus, a more selective tracer is needed to identify the early stages of reactive fibrosis in HF with pressure overload and remodeling. Fibroblast activation protein (FAP) is a specific marker of activated fibroblasts, whilst FAP is not expressed by inactive fibroblasts or fully differentiated myofibroblasts and non-fibroblast cells. The tracer of radiolabeled FAP inhibitor (FAPI) accumulates intensely in the territory of an MI, as identified by decreased [^18^F]FDG uptake and confirmed by CMR and microscopy [68]. The advantage over [^18^F]FDG imaging is the lack of background activity in FAP-targeted imaging, which provides high image contrast. The studies on FAPI are mainly in the field of post-MI and ischemic HF, whereas the application of FAPI in nonischemic HF is less well characterized and relies predominantly on animal models [16,17,18].

### 2.4. Sympathetic Denervation

The autonomic nervous system plays a significant role in the regulation of heart rate, blood flow, and contractility, but it becomes dysfunctional in patients with HF [69]. Innervation imaging research using [^123^I]metaiodobenzylguanidine SPECT has demonstrated that sympathetic nervous system activation leads to the downregulation of beta-adrenergic receptors and promotes LV remodeling in HF patients [27]. Ischemic sympathetic nerve damage has also been observed in patients with stable coronary disease, most likely resulting from a combination of neuronal stunning, decreased cell function, and anatomical denervation. In patients with MI, both nerve terminals and nerve fibers within the ischemic zone are damaged, and denervation often extends beyond the scar in transmural MI [70]. The term “sympathetic denervation” should be reserved for situations where there is an anatomic loss of sympathetic nerves, whereas “dysinnervation” or sympathetic “dysfunction” can be used as a general term where the relative contributions of reversible neuronal stunning or anatomic denervation are not known [70,71].

Cardiac innervation can be visualized using several PET tracers, such as [^11^C]hydroxyephedrine ([^11^C]HED), [^11^C]epinephrine, and [^18^F]fluorohydroxyphenethylguanidines. ^11^C-HED is the most routinely used PET imaging agent for sympathetic nerve activity and binds to the presynaptic uptake-1 transporter at sympathetic nerve endings [72]. Regional sympathetic denervation assessed by [^11^C]HED retention index was correlated with decreased systolic wall thickening and an increase in late gadolinium enhancement (LGE) in patients with HFpEF [20]. In patients with severe ischemic HF, sympathetic denervation identified by [^11^C]HED PET was a significant predictor of sudden cardiac death, independent of LVEF and MI size [71]. Moreover, integration of [^18^F]FDG PET/CT-derived 3D scar maps could facilitate substrate-based ventricular tachycardia ablation by identifying non-transmural scar undetectable by endocardial voltage recordings [73]. More recent investigations have provided some evidence of the utility of [^11^C]HED in predicting responses to cardiac resynchronization therapy (CRT) in eligible patients (see “Clinical Applications”).

## 3. Clinical Applications

In the field of HF, different imaging techniques are used in daily practice to guide diagnosis, risk stratification, and treatment [74]. Whereas echocardiography remains the frontline modality for initial assessment, the role of computed tomography, CMR, and nuclear imaging continues to expand (Table 2) [75]. A detailed review of techniques for multimodality imaging in HF is beyond the scope of this paper. Thus, we will focus on different applications of PET imaging in patients with HF in the following paragraphs.

### 3.1. Prognosis and Risk Stratification

In patients with HF and known or suspected CAD, PET myocardial perfusion imaging is considered the most sensitive imaging technique to predict recovery of LV dysfunction [77,78,79] and it has also emerged as a sensitive prognostic tool [3,80]. Reduced MPR as assessed by PET in patients with ischemic HF was shown to be associated with major adverse cardiac events [79] with a higher sensitivity than reduced LVEF in predicting cardiac death [81]. Furthermore, PET can be used to assess viability before revascularization in ischemic cardiomyopathy. Even though the large PARR2 clinical trial that investigated the role of viability imaging before revascularization using PET was negative, subsequent analysis of the trial suggested a clinical benefit when adhering to the PET results [82]. Both European and American HF Guidelines state that PET may be considered for the assessment of myocardial ischemia and viability in patients with CAD who are considered suitable for coronary revascularization (class IIb) [1,83]. 

Patients with dilated cardiomyopathy have reduced MBF and reduced coronary flow response to sympathetic stimulation, which carries a poor prognosis [84,85]. Patients with hypertrophic cardiomyopathy have a higher risk of cardiac death or worsening HF with progressive reduction of hyperemic flow and myocardial flow reserve [86]. Overall, there is consistent evidence of the value of PET imaging as a tool able to provide prognostic risk stratification. 

### 3.2. Response to Heart Failure Therapy

The treatment of HFrEF is based on established guideline-directed therapy, both medical and with devices (i.e., CRT). Hasegawa et al. [87] evaluated myocardial [^18^F]FDG PET at baseline in patients with dilated cardiomyopathy, who were then started on beta-blocker therapy. The uptake of [^18^F]FDG after glucose loading was a good predictor of the response to beta-blockers. The [^18^F]FDG uptake patterns during fasting and after glucose loading provided some indication of the histologic findings, since they were correlated with the presence of fibrosis, contractility failure, and muscle bundle fragmentation [87]. 

Cardiac resynchronization therapy (CRT) is an effective therapy for HFrEF and leads to improved quality of life and reductions in HF hospitalization rates and all-cause mortality [88,89,90]. The first studies with PET imaging in CRT patients investigated the effects of CRT on perfusion and metabolism, both on global and regional levels [15]. Among studies assessing MBF, CRT seems not to produce significant effects on global myocardial perfusion [15,91,92,93,94,95,96]. On the other hand, the regional metabolic heterogeneity, which is typical in patients with HF and left bundle branch block, was nearly normalized by CRT [95,97]. Typically, the septal wall metabolism is clearly reduced, and lateral wall metabolism increased, leading to abnormal septal-to-lateral wall ratios of MVO_2_ and MBF [98]. Following CRT treatment, there is a decrease in MVO_2_ and MBF in the lateral wall, together with an increase in the septum, which results in a more uniform distribution [15]. Furthermore, several studies showed that an increase in cardiac work with CRT is not associated with an increase in MVO_2_ [15,93,94,99,100].

Besides metabolism and MBF, neurohormonal modulation also plays a role in CRT-driven improvement of LV function [101]. [^11^C]HED PET imaging has been used to analyze cardiac innervation in patients with CRT [102,103]. Patients with dilated cardiomyopathy, LVEF ≤ 35%, and NYHA class II or III underwent [^11^C]HED PET prior to and early (i.e., 1 week and 3 months) after CRT implantation [102]. Compared to non-responders, patients that responded better to CRT therapy had higher [^11^C]HED uptake and less regional heterogeneity in tracer uptake at baseline. Moreover, responders had significant improvements in total myocardial [^11^C]HED uptake and regional heterogeneity. In partial agreement, another study of a similar design showed that regional heterogeneity but not global myocardial [^11^C]HED uptake improved 3 months after CRT implantation in more severe (NYHA class III and IV) HF patients [103]. Together, these studies support the hypothesis that after CRT implantation there is an improvement in sympathetic presynaptic function. However, they are based on an arbitrary definition of “CRT response”, which is controversial since patients who do not fulfill “CRT response” in terms of LV function or symptoms may well have experienced the mortality benefit [1,88]. In any case, nuclear imaging should not guide patient selection for CRT, since the latter relies on guideline-based criteria based on symptoms, QRS duration, and QRS morphology [1].

### 3.3. Infiltrative Cardiomyopathies

Infiltrative cardiomyopathies are caused by the abnormal deposition of specific substances in the heart, leading to impaired cardiac function and HF [104].

Cardiac amyloidosis, most commonly due to acquired light-chain (AL) or transthyretin-related (ATTR) amyloidosis, is characterized by extracellular deposition of amyloid fibrils within the heart and can manifest as clinical HF. Whilst Technetium-99m-pyrophosphate ([^99m^Tc]PYP) scintigraphy is the preferred diagnostic exam for ATTR amyloidosis, there are several amyloid-binding PET tracers used in cerebral amyloidosis imaging that could also detect cardiac amyloidosis and possibly be useful in monitoring the response to disease-specific treatments (e.g., Tafamidis). These tracers include [^18^F]florbetaben, [^18^F]florbetapir, and [^11^C]Pittsburg compound B ([^11^C]PiB) [27]. Pilot studies using PET/CMR with [^18^F]NaF or PET/CT with [^18^F]florbetaben showed that these techniques can discriminate ATTR from AL amyloidosis and control subjects without the disease [105,106]. Another small study using a combination of [^99m^Tc]PYP scintigraphy and [^11^C]PiB PET resulted in a good differentiation between ATTR and AL amyloidosis [107]. A recent study on 41 chemotherapy-naïve AL cardiac amyloidosis patients revealed that [^11^C]PiB uptake reflects the degree of myocardial amyloid load and is an independent predictor of clinical outcome [108]. However, more large-scale data are needed to compare the diagnostic and prognostic performance of different PET tracers in cardiac amyloidosis compared to bone scintigraphy and CMR.

Anderson–Fabry disease is an x-linked lysosomal storage disorder caused by defective activity of alpha-galactosidase A, a lysosomal enzyme, resulting in the amassment of globotriaosylceramide in lysosomes in multiple cell types throughout the body [2,109]. The cardiac manifestation is an infiltrative cardiomyopathy ultimately causing HF, which may be preventable with enzyme replacement therapy [110,111,112,113]. Thus, early diagnosis of cardiac involvement is important. Currently, CMR and, more rarely, endomyocardial biopsy are considered to be the gold standard to diagnose cardiac involvement in Anderson–Fabry disease [114]. In a study using [^18^F]FDG PET/CMR hybrid imaging to assess cardiac involvement in asymptomatic patients with Anderson–Fabry disease, all patients with LGE and edema showed focal FDG uptake in the corresponding myocardial segments, indicating inflammation [115]. This supports the role of inflammation in the pathogenesis of cardiac involvement in Anderson–Fabry disease and demonstrates the possibility of evaluating different disease stages using multimodality PET/CMR [2].

### 3.4. Cardiac Sarcoidosis

Recent guidelines have been published guiding the use of [^18^F]FDG PET for the assessment of patients with sarcoidosis and suspected cardiac involvement [23]. The rationale of [^18^F]FDG PET in cardiac sarcoidosis is that active inflammatory cells in sarcoid granulomas are characterized by increased glucose metabolism, therefore, they avidly take up glucose and its analogs [116]. Cardiac PET is usually combined with whole-body CT imaging to uncover extracardiac involvement [117]. In normal conditions, the myocardium may be a site of physiological [^18^F]FDG uptake due to the glucose consumption of myocardial cells. To discriminate among physiological [^18^F]FDG uptake in the myocardium and abnormal [^18^F]FDG uptake due to myocardial inflammation, for diagnostic imaging, it is recommended to use patient preparation protocols (Table 3), including a low-carbohydrate/high-fat diet followed by fasting and, in some centers, by additional intravenous unfractionated heparin to raise the availability of fatty acids, although the contribution of heparin is unclear [23,76]. Regardless, 10%–15% of cardiac PET studies are diagnostic failures due to poor suppression of physiologic glucose uptake [118]. Thus, PET findings must be incorporated into a multiparametric diagnostic pathway established by a combination of pathology, imaging, and clinical and/or electrocardiographic findings [24,119,120].

[^18^F]FDG PET scanning in cardiac sarcoidosis also carries therapeutic and prognostic implications. Initiating immunosuppression in these patients presupposes proof of inflammatory activity. A study on 96 patients with cardiac sarcoidosis who performed [^18^F]FDG PET prior to starting immunosuppressive therapy showed that pre-treatment myocardial uptake had a strong positive correlation with a change in LVEF following immunosuppression [121]. Furthermore, repeat scans after treatment initiation may help identify responses to, and relapses after, therapy, thus guiding titration of immunosuppressive therapy to improve or prevent HF [122,123]. 

The prognostic value of PET was confirmed in a recent meta-analysis [124], though not all works are supportive [125,126]. Major challenges in cardiac sarcoidosis remain, particularly the lack of a simple gold standard, the often delayed diagnosis, sampling error when facilitating endomyocardial biopsy, and the potential overlap with other cardiomyopathies that may present a “hot phase”, which make the diagnosis often difficult [127,128,129,130]. In the future, cardiac PET studies can involve tracers that work without dietary preparation, such as somatostatin analogs, and hybrid PET/CMR imaging may improve diagnostic accuracy [23,131,132].

**Table 3:** Different dietary protocols to achieve the best myocardial suppression for cardiac sarcoidosis diagnosis with [^18^F]FDG-PET, according to Özütemiz et al.[133]. Notably, the 72-h daytime ketogenic diet with 3-days overnight fasting (diet C) achieved substantially superior myocardial suppression versus a 24-h ketogenic diet with overnight fasting (diet A) and an 18-h fast (diet B).

Diet	Description
A	High-fat, low-carbohydrate diet beginning 24 h before the study. Nothing by mouth, except water and oral pills, 6 h before the examination.
B	18 h of fasting before the study. Nothing by mouth, except water and oral pills, for 18 h before the study.
C	High-fat, low-carbohydrate ketogenic diet beginning 72 h before the study. Nothing by mouth, except water and oral pills, for 3 overnight fasts before the exam. First 2 nights from 08:00 PM until at least 08:00 AM the next morning. The night before the test from 08:00 PM until the time of the test. For diabetics, consider avoiding prolonged fasting, preferably complete the examination during afternoon hours after ketogenic breakfast and morning insulin, followed by 6-h fasting.

### 3.5. Cardio-Oncology

Successful cancer treatment can be hindered by the risk of cardiotoxicity, which can manifest as subclinical LV systolic dysfunction and even overt HF [134]. Although the gold standard for risk stratification and follow-up remains echocardiography [135], there are some emerging tracers for detecting early cardiotoxicity by PET imaging.

Cardiac function depends on energy production, and the metabolic pathways that drive this process are promising for early identification of cardiotoxicity. Non-invasive visualization of a compensatory increase in glycolysis, induced by a reduction in β-oxidation, can be achieved with [^18^F]FDG PET. Increased glucose utilization, indicated by increased [^18^F]FDG uptake, has been observed in rat hearts after therapy with sunitinib, a multi-targeted receptor tyrosine kinase inhibitor [43]. Furthermore, cardiac uptake of [^18^F]FDG increases in patients with lung cancer and esophageal cancer treated with radiotherapy and with lymphoma treated with anthracyclines [136,137,138].

^11^C-labelled tracers for the study of myocardial metabolism are potential alternatives to [^18^F]FDG because they are fully metabolized by the heart and allow for qualitative estimation of the rates of metabolic processes. In preclinical models of sunitinib cardiotoxicity, a decreased rate of myocardial ^11^C-acetate utilization has been observed [43]. Similarly, in rats with doxorubicin-induced cardiotoxicity, the myocardium does not utilize [^11^C]acetoacetate, a ketone body, to the same extent as healthy controls [139]. However, the clinical application of these ^11^C-labelled tracers is currently limited by the need for complex metabolite analysis to derive a suitable input function for kinetic modeling [140]. Interestingly, a fully automated method for estimating myocardial external efficiency based on a [^11^C]-acetate PET without ECG-gating has been recently developed [141].

The most common cardiac complication during therapy with immune checkpoint inhibitors (ICI) is myocarditis [142], in which early detection and diagnosis are crucial. In a recent study, the use of [^68^Ga]DOTATOC PET/CT along with immune correlates is a highly sensitive method to detect ICI-related myocarditis, especially in the early stage of myocardial inflammation, as patients with elevated troponin may present normal CMR imaging results [143]. Another retrospective study of [^68^Ga]FAPI PET/CT showed that patients developing ICI-related myocarditis had cardiac enrichment of the tracer, which was less distinct or absent in patients receiving ICIs without any signs of immunological adverse effects or cardiac impairment [144].

Anthracycline-induced cardiotoxicity mainly relies on mitochondrial anthracycline accumulation in the cardiomyocytes and disruption of mitochondrial structure and function [145]. A recent study highlights the possibility of visualizing disrupted mitochondrial membrane potential by PET in a rat model of doxorubicin-induced cardiotoxicity [146]. Future clinical evaluations are required to determine the sensitivity of this technique to anthracycline-induced cardiomyopathy in humans.

## 4. Future Directions

The future of PET imaging in HF will rely on the use of novel tracers to discover the latest biological processes, and on new possible clinical applications. PET can be included in clinical trials to assess the response of novel therapies in the pathophysiology of HF. In the field of cardiomyopathies, the use of novel tracers targeted to specific pathways and receptors, such as myocardial angiotensin II receptor type 1 [147], could shed further light on potential cardiomyopathic mechanisms. Despite the potential benefits of PET imaging in the assessment of HF, it is not yet widely available and is mainly restricted to specialized centers. Further studies are needed to establish the role of PET imaging in the management of HF and to determine its cost-effectiveness compared to other imaging modalities. Nonetheless, PET functional imaging represents a promising avenue for improving the diagnosis, treatment, and management of HF.

## Figures and Tables

**Table 1:** Overview of most used PET tracers in functional cardiovascular imaging.

Tracer	Name	Function
H_2_[^15^O]	H_2_^15^O-water	Myocardial blood flow quantification (gold standard) [5]
^82^Rb	Rubidium-82	Myocardial blood flow quantification [6]
[^13^N]H_3_	^13^N-Ammonia	Myocardial blood flow quantification [7,8,9,10]
^[18^F]Flurpiridaz	^18^F-Flurpiridaz	Myocardial blood flow quantification [11]
[^11^C]Acetate	Carbon-11 labeled acetate	Myocardial blood flow quantification, myocardial oxygen consumption, and cardiac efficiency [12,13,14,15]
[^68^Ga]FAPI (i.e., FAPI-04, FAPI-46, etc.)	Gallium-68 labeled fibroblast activation protein inhibitor	Detection of fibrosis through targeting activated fibroblast response [16,17,18,19]
[^18^F]AlF-NOTA-FAPI-04	Aluminum-[^18^F]Fluoride labeled fibroblast activation protein inhibitor-04 chelated with NOTA	
[^11^C]HED	Carbon-11 labeled hydroxyephedrine	Global and regional sympathetic innervation quantification [20,21]
[^18^F]FDG	Fluorine-18 labeled fluorodeoxyglucose	Evaluation of increased metabolism (e.g., inflammation) [2,22,23,24] and myocardial viability

**Table 2:** Overview of general indications and applications of cardiac PET imaging according to the ESC and EACVI/EANM recommendations [1,76]. CIED, cardiac implantable electronic devices; LVAD, left ventricular assist devices.

Indication	Purpose
Coronary artery disease	Viability assessment before revascularization
Cardiac sarcoidosis	Diagnosis; treatment monitoring
Cardiac amyloidosis	Diagnosis; research
Acute myocarditis	Additional diagnostic test
Prosthetic valve endocarditis	Diagnosis
Infection of CIED	Diagnosis; infection extent
Infection of LVAD	Diagnosis; infection extent
Myocardial innervation	Mainly research

## Data Availability

Not applicable.
